# Real-life effectiveness of budesonide/formoterol maintenance and reliever therapy in asthma patients across Asia: SMARTASIA study

**DOI:** 10.1186/1471-2466-13-22

**Published:** 2013-04-04

**Authors:** Nanshan Zhong, Jiangtao Lin, Parthiv Mehta, Pintip Ngamjanyaporn, Tzu-Chin Wu, Faisal Yunus

**Affiliations:** 1 Respiratory Research Institute, 1st Affiliated Hospital of Guangzhou Medical College, Guangzhou, China; 2 China-Japan Friendship Hospital, Beijing, China; 3 Mehta’s Hospital and Cardiopulmonary Care Centre, Gujarat, India; 4 Allergy Immunology and Rheumatology Division, Department of Medicine, Faculty of Medicine, Ramathibodi Hospital, Mahidol University, Bangkok, Thailand; 5 Chung Shan Medical University Hospital, Taichung, Taiwan; 6 Department of Pulmonology and Respiratory Medicine, Faculty of Medicine, University of Indonesia, Persahabatan Hospital, Persahabatan, Indonesia

**Keywords:** Asthma management, Asthma control, Budesonide/formoterol, Symbicort maintenance and reliever therapy

## Abstract

**Background:**

The use of budesonide/formoterol in a single inhaler for both maintenance and reliever therapy is a recommended option for treatment of persistent asthma not responding well to inhaled corticosteroid (ICS) alone.

**Methods:**

This was a multi-centre open-label study on patients whose asthma condition remained inadequately controlled by various asthma treatments other than budesonide/formoterol. After a 2-week run-in period, eligible patients underwent a 12-week treatment period with budesonide/formoterol (Symbicort SMART®, 160/4.5 μg) twice daily plus as needed. Patient’s asthma control and quality of life were assessed using the 5-item Asthma Control Questionnaire (ACQ-5) and the standardized Asthma Quality of Life Questionnaire (AQLQ-S), respectively.

**Results:**

A total of 862 eligible asthma patients who have had asthma for a mean duration of 10.73 ± 12.03 years entered a 12-week treatment with budesonide/formoterol maintenance and reliever therapy. During treatment, ACQ-5 score improved significantly by 0.58 ± 0.93 (95% CI, 0.51 to 0.64, P < 0.0001) from the baseline level of 1.62 ± 1.00. AQLQ(S) score improved by 0.70 ± 0.89 (95% CI, 0.64 to 0.76, P < 0.0001) from baseline. Asthma symptom score was also reduced significantly (P < 0.0001); between run-in and treatment periods, night- and day-time symptom scores were reduced by 0.32 ± 0.54 (95% CI, 0.28 to 0.35) and 0.30 ± 0.52 (95% CI, 0.27 to 0.34), respectively. The percentage of nights with awakenings due to asthma symptoms was reduced by 11.09 ± 26.13% (95% CI, 9.34 to 12.85%), while the percentage of asthma-control and symptom-free days increased by 20.90 ± 34.40% (95% CI, 18.59 to 23.21%) and 23.89 ± 34.62% (95% CI, 21.56 to 26.21%), respectively (P < 0.0001). Together with the improvement in asthma control, the number of night- and day-time inhalations of as-needed reliever medication decreased by 0.30 ± 0.82 (95% CI, 0.24 to 0.35) inhalations and 0.30 ± 0.97 (95% CI, 0.23 to 0.36) inhalations, respectively (P < 0.0001). No unexpected adverse events were reported.

**Conclusion:**

During treatment of inadequately controlled asthmatic patients with budesonide/formoterol maintenance and reliever therapy, significant improvement in patients’ asthma control and reductions in asthma symptoms and as-needed medication use was observed. Patients’ quality of life was improved and the treatment was well tolerated.

**Trial registration:**

ClinicalTrial.gov: (NCT00939341)

## Background

Inhaled corticosteroid (ICS) is the first-line treatment for the management of asthma not controlled by on-demand short-acting β_2_ agonists (SABA), and is the mainstay of asthma therapies today [1,2]. A SABA is often prescribed with ICS for relief of occasional breakthrough symptoms during ICS treatment, but overuse of SABA for quick relief of asthma symptoms has been associated with poor asthma control [3]. Achieving and maintaining asthma control with the minimal effective dose of medication(s) used is the goal of all asthma therapies. But in reality, a large proportion of patients undergoing various asthma treatments considered conventional best practices fail to achieve and/or maintain full asthma control [4,5]. Complexity of treatment regimens, poor adherence and flawed inhalation techniques are known factors contributing to suboptimal asthma treatment outcomes. For adult patients whose asthma remains uncontrolled despite regular inhalation of a low-to-medium dose of ICS, current clinical practice guidelines recommend a step-wise approach that is based on patient’s attainment of his/her asthma control, which might include increasing the existing low-dose ICS inhalation or adding on to low-dose inhaled ICS therapy a long-acting β2 agonist (LABA) for control of lung function and symptoms, and to reduce the risk of exacerbations, and optionally, an additional SABA for as-needed rapid relief of breakthrough symptoms in poorly controlled cases [6,7]. If this approach fails to control the asthma, a further stepwise addition of a leukotriene receptor antagonist (LTRA) or theophylline could be considered. Clinical evidence suggests that the addition of a LABA could provide better asthma control than the mere upward titration of the patient’s ICS dose [8]. Budesonide/formoterol in a single inhaler for both maintenance and reliever therapy is now an established therapeutic option for management of inadequately controlled asthma. This single inhaler budesonide/formoterol maintenance and reliever therapeutic strategy is an approach recognised by regulatory authorities in many countries and is now recommended by the Global Initiative for Asthma (GINA) for treatment of adult patients with uncontrolled asthma, which GINA defines as having an exacerbation or at least three of the following in a given week: daytime symptoms >2 times/week, any limitation of activities, any nocturnal symptoms/awakenings, need for reliever treatment >2 times/week or lung function <80% predicted normal [6]. In controlled clinical trials, the single inhaler budesonide/formoterol maintenance and reliever therapeutic approach has been shown to be more effective than conventional best practices in achieving asthma control, with a safety profile that is similar to treatment with ICS alone administered at higher doses [9-14]. A recent post-hoc analysis of five large clinical trials (>12000 patients) comparing budesonide/formoterol single inhaler maintenance and reliever therapy with other treatments shows it as a highly effective option for patients requiring treatment adjustments across Steps 2 to 4 in the GINA treatment guidelines [15]. However, other than the evidence obtained from clinical trials, there is a paucity of information on the effectiveness of budesonide/formoterol maintenance and reliever therapy in real-life clinical practice. This study was designed to assess in a real-life setting the effectiveness of the single inhaler budesonide/formoterol maintenance and reliever therapy (Symbicort SMART®) in the management of asthma inadequately controlled by patients’ prior other asthma medications.

## Methods

### Study design

This was a 12-week multi-centre open-label therapeutic Phase IV study designed to evaluate the effects of a single inhaler budesonide/formoterol maintenance and reliever therapy on asthma patients in 5 countries/areas across Asia whose asthma condition remained inadequately controlled despite having been on continuous treatment with various asthma therapies, except budesonide/formoterol, for at least 4 weeks. The study was designed to mimic as much as possible routine clinical practice to minimise interference with physicians’ and patients’ behaviours that might influence outcomes.

### Study population and sample size

The target population were patients aged 18 years and above with confirmed diagnosis of asthma as defined by GINA 2007 guidelines [16], who have had asthma for at least 6 months, were on continuous asthma treatment within the 4 weeks preceding screening and whose asthma condition was partly controlled or uncontrolled. The eligible patient must have demonstrated reversible airway obstruction, defined as an increase in FEV_1_ ≥12% and 200 ml from pre-bronchodilator value. Patients were excluded if they were found to have any of the following: chronic obstructive pulmonary disease; previous treatment with budesonide/formoterol; current use of any β–blocker therapy; use of ICS within the 30 days preceding this study enrolment; or a smoking history of ≥10 pack-years. Additionally, to enter the study treatment period, patient must have experienced no asthma exacerbation during the run-in to treatment.

### Study objectives

The primary objective of the study was to compare, at a regional level, the effects of inhaled budesonide/formoterol maintenance and reliever therapy on partially controlled or uncontrolled asthma patient with the patient’s previous therapy by assessing the changes in the Asthma Control Questionnaire (ACQ-5) score. The secondary objectives of the study were: (i) to document the well-being of patients using budesonide/formoterol maintenance and reliever therapy in different countries/areas within the general practice setting, by assessing the change in ACQ-5 score from baseline at country/area level; (ii) to assess the clinical effectiveness of budesonide/formoterol maintenance and reliever therapy on patients’ quality of life, by change in standardized Asthma Quality of Life Questionnaire (AQLQ-S) and overall scores from baseline at regional level; and (iii) to document the usage and compliance, and also patient’s perception of budesonide/formoterol maintenance and reliever therapy within general practice setting. Study medication use including mean number of total and as-needed inhalations per day, number (%) of patients taking more than a total of 12 inhalations on at least 1 day and number (%) of patients taking at least 9 inhalations in total every day for more than 2 weeks were documented.

### Study interventions, procedures and outcome measures

**Figure 1:**
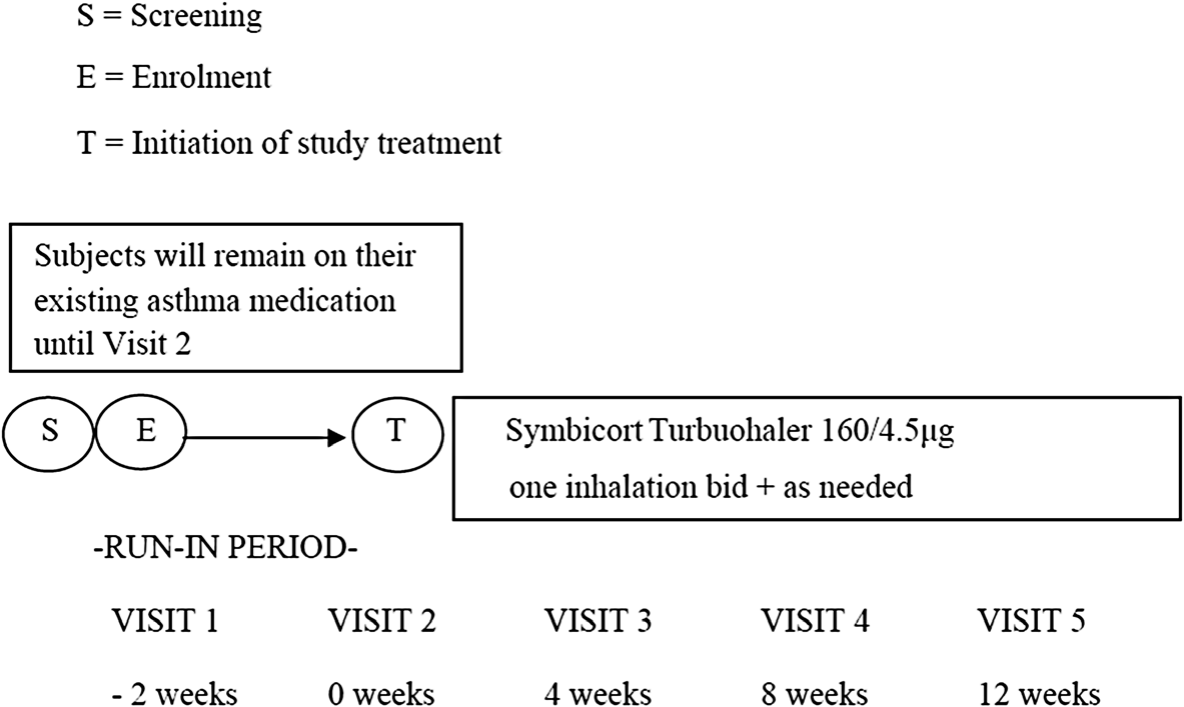
Study flow chart.

The study flow is depicted in Figure 1. Eligible patients went through a 2-week run-in period during which time they were kept on their existing asthma medications, followed by a switch to a 12-week treatment with a single inhaler therapy containing budesonide (160 mg) and formoterol (4.5 mg) (budesonide/formoterol; Symbicort SMART®, 160/4.5; AstraZeneca, Lund, Sweden), during which time, patients self-administered a one inhalation, twice daily regimen of the budesonide/formoterol, with one inhalation in the morning and one inhalation in the evening, plus as-needed additional inhalations in response to symptoms. Patients’ asthma control and quality of life were assessed using the validated 5-item ACQ-5 and the standardized AQLQ-S, respectively [17-19]. These assessments were made at baseline (Visit 2) and then at Visit 3, four weeks into the treatment, Visit 4, eight weeks into the treatment, and again at Visit 5, twelve weeks into the treatment. For both the ACQ-5 and AQLQ-S, a minimal important difference (MID) for clinical significance was set at 0.5 unit change in score from baseline level [20]. To assess lung function, patient’s forced expiratory volume in one second (FEV_1_) was measured at baseline (Visit 2), just before the initiation of treatment with Symbicort maintenance and reliever therapy, and at Visit 3, four weeks into the treatment, and again at Visit 5, twelve weeks into the treatment. FEV_1_ was measured using a standard spirometry method [21]. Throughout the study duration, the patient recorded on a diary card the following outcome measures: (i) asthma symptom score, day and night; (ii) nights with awakening(s) due to asthma symptoms; and (iii) number of inhalations of budesonide/formoterol. Post-treatment changes in these parameters were then evaluated with reference to the pre-treatment (baseline) levels.

Prior to commencement of this study, approvals to conduct the study were obtained from the appropriate independent ethics committee and/or institutional review boards governing the respective participating centres in each country/area (refer to full list under Appendix I). The study was performed in accordance with the principles of the International Conference on Harmonisation (ICH) Harmonised Tripartite Guideline for Good Clinical Practice (GCP). Written informed consent was obtained from each participating patient before he/she proceeded to participate in the study.

### Evaluation of safety

Safety data were collected throughout the study duration. Adverse events, serious adverse events and discontinuations due to adverse events were recorded as when reported by study investigators and coded according to MedDRA (Version 12.0). Safety evaluation included the data collected from all patients who took at least one dose of the investigational drug during the treatment period, and for whom safety data have been collected post-initiation of the study treatment.

### Statistical methods

The study population size was estimated according to the precision with which the effect size is set to be reported. Based on the primary variable of this study, which is the mean change in ACQ-5 score from baseline level, we determined a sample size of about 1000 patients in the entire Asian region to give us a 95% confidence interval (CI) with an approximate width of 0.12 units when standard deviation (SD) is 1.0 unit. All statistical analyses were performed on an intention-to-treat basis, using data from all participating patients who had taken at least one dose of the investigational drug and had data collected after initiation of the drug treatment. The primary outcome variable, change in ACQ-5 score from baseline to treatment period, was analysed using paired *t*-test, with significance set at the 5% level. A change of ≥0.5 in the score was deemed clinically important. The changes in AQLQ-S overall and domain scores were analysed in a similar way. G-mean FEV_1_ was derived via a multiplicative analysis of variance with patient, period and treatment set as fixed factors. FEV_1_ and all other secondary patient-reported outcomes were analysed as change from baseline level using paired *t*-test, at the 2-sided 5% level of significance. Safety data were analysed using descriptive statistical methods.

## Results

### Patients’ demographics and baseline characteristics

Between July 2009 and August 2010, a total of 1022 eligible asthma patients were enrolled from 51 study centres across China, India, Indonesia, Thailand and Taiwan. Amongst them, 862 (84.3%) patients entered into the study treatment period. There were 407 males and 455 females with mean age (± SD) of 44.7 ± 13.7 years. Together, they have had asthma for a mean duration of 10.73 ± 12.03 years, and the majority of these patients (69%) were being treated with Salbutamol (393 patients; 45.6%) or combination of Fluticason and Salmeterol (202 patients; 23.4%), while the rest were treated with various other asthma medications. Most patients were also on concomitant ICS. At entry to this study, 719 patients (83.4%) were on ICS, with 525 patients (60.9%) on LABA plus ICS, and 135 patients (15.7%) on SABA plus ICS.

Of the patients who entered into the study treatment period, 66 (7.7%) of them discontinued from the study for various reasons, which include adverse events (15 patients), pregnancy (1 patient), self-withdrawal (13 patients), deviations from study protocol (6 patients), incorrect enrolment (8 patients) and lost to follow-up (23 patients). The 862 patients who took at least one dose of the investigational drug, budesonide/formoterol, formed the set of subjects for analysis of both the efficacy and safety outcomes of this study (Table 1). The demographic and baseline characteristics of the subjects in the full analysis set are summarised in Table 2.

**Table 1:** Study population (Full analysis set)

	**China**	**India**	**Indonesia**	**Taiwan**	**Thailand**	**All**
Patients in full analysis set	407	162	61	103	129	862
Patients in safety analysis set	407	162	61	103	129	862

**Table 2:** Demographic and baseline characteristics of patients

**Demographic characteristic**		**Number (%) of patients N = 862**
Sex	Male	407 (47.2)
	Female	455 (52.8)
Age (years)	n	862
	Mean (SD)	44.7 (13.7)
	Range	18 - 81
Race	Asian	862 (100.0)
BMI (kg/m**2)	n	862
	Mean (SD)	24.36 (4.10)
	Range	14.2 - 47.5
Time since diagnosis (years)	n	862
	Mean (SD)	10.73 (12.03)
	Range	0.1 - 60.0
Smoking	Non smoker	744 (86.3)
	Ex-smoker	84 (9.7)
	Occasional smoker	18 (2.1)
	Habitual smoker	16 (1.9)
Pack-years	n	116
	Mean (SD)	4.8 (2.9)
	Range	0 - 15
Inhaled GCS at entry	No	143 (16.6)
	Yes	719 (83.4)
Inhaled GCS at entry: dose (ug/day)	n	317
	Mean (SD)	557.8 (321.5)
	Range	50 - 1600
FEV1 (L)	n	862
	Mean (SD)	2.063 (0.709)
	Range	0.69 - 4.63
FEV1% of predicted normal *	n	862
	Mean (SD)	70.38 (17.37)
	Range	22.4 - 124.3
FEV1% Reversibility	n	862
	Mean (SD)	26.50 (15.15)
	Range	9.8 - 139.5

^a^ FAS: All enrolled subjects except for screening failure.

* Predicted normal FEV1 value (post-bronchodilator) was calculated according to ERS [22].

### Assessments of impact on patients’ asthma control

#### Change in ACQ-5 score from baseline at regional level

**Figure 2:**
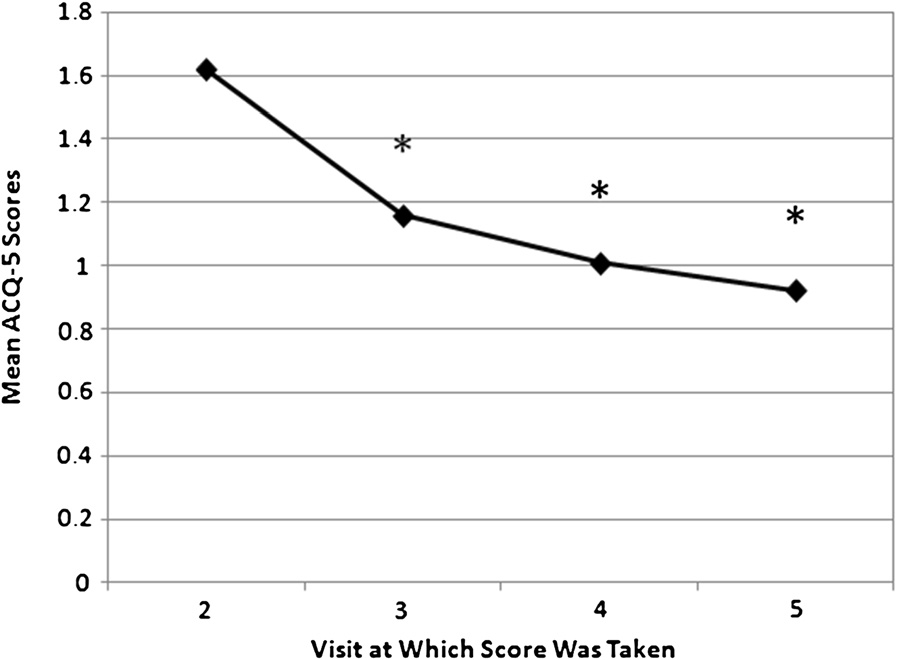
**Mean ACQ-5 scores following initiation of treatment with budesonide/formoterol maintenance and reliever therapy (regional level).** * denotes significant difference from baseline level. Visit 2 = baseline.

At the regional level, patients’ asthma control significantly improved during treatment with budesonide/formoterol maintenance and reliever therapy. This is reflected by the consistent reduction in mean overall ACQ-5 score from baseline to 4, 8, and 12 weeks of the therapy (Figure 2 and Table 3). A significant decrease in ACQ-5 score was evident from 4 weeks post-initiation of treatment with budesonide/formoterol maintenance and reliever therapy, indicating early onset of improvement in asthma control. During the treatment period, overall mean ACQ-5 score improved significantly by 0.58 ± 0.93 (95% CI, 0.51 to 0.64; P < 0.0001). Clinically important improvement in ACQ-5 score (change ≥ MID of 0.5) was observed by 8 weeks of treatment. During treatment, 48.2% of the patients experienced improvement in symptoms, while only 8.8% experienced worsening of symptoms.

**Table 3:** Summary of mean values of overall ACQ-5 score at a regional level

**Variable**	**Visit**	**Observed value**			**Change from visit 2 (baseline)**			**95% CI**		**Paired *t*-test**
		**N**	**Mean**	**SD**	**N**	**Mean**	**SD**	**Lower**	**Upper**	**p-value**
Overall score	2	854	1.62	1.00	-	-	-	-	-	-
	3	833	1.16	0.88	826	−0.46	0.96	−0.52	−0.39	<.0001
	4	803	1.01	0.85	796	−0.60	1.01	−0.67	−0.53	<.0001
	5	794	0.92	0.85	787	−0.69	1.09	−0.77	−0.62	<.0001
	Mean of 3-5	841	1.04	0.75	834	−0.58	0.93	−0.64	−0.51	<.0001

#### Change in ACQ-5 score from baseline at country/area level

**Figure 3:**
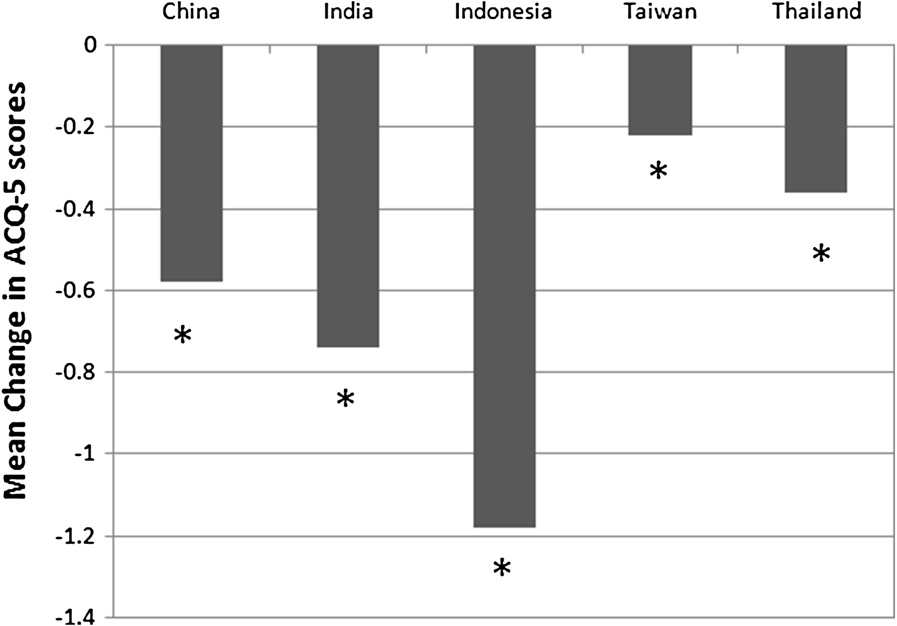
**Mean Change in ACQ-5 scores from baseline to treatment period (by country/area).** * denotes significant difference from baseline level.

Significant reduction in mean ACQ-5 score of patients was observed in all 5 participating countries during the period they were treated with budesonide/formoterol maintenance and reliever therapy (P < 0.0001 to P = 0.0089), indicating significant improvements in asthma control across the region during the treatment (Figure 3 and Table 4). Statistically significant changes were observed from as early as 4 weeks after initiation of the treatment. However, the quantum of change in ACQ-5 scores differed significantly between study populations in the various countries/areas (P < 0.0001); the change was highest amongst the study population in Indonesia, followed by that of those in India, China, Thailand and Taiwan. Improvement in asthma control reached clinical importance (change in ACQ-5 score ≥ MID of 0.5) only in the study populations in China, India and Indonesia; in China and Indonesia, clinically important improvement in ACQ-5 score was achieved by 4 weeks post-initiation of treatment with budesonide/formoterol maintenance and reliever therapy.

**Table 4:** Summary of period means of overall ACQ-5 score for mean of visit 3–5 at country/area level

**Overall score**		**N**	**Visit 2 (baseline)**		**Mean of visit 3-5**		**Mean change**	**95% confidence interval**		**Paired *t*-test**
			**Mean**	**Range**	**Mean**	**Range**		**Lower**	**Upper**	**p-value**
Country or Area	China	386	1.72	0.0 - 5.0	1.14	0.0 - 4.4	−0.58	−0.67	−0.49	<.0001
	India	159	1.87	0.0 - 5.4	1.12	0.0 - 3.3	−0.74	−0.92	−0.57	<.0001
	Indonesia	61	1.78	0.0 - 3.8	0.61	0.0 - 2.9	−1.18	−1.40	−0.95	<.0001
	Taiwan	100	1.11	0.0 - 4.8	0.89	0.0 - 2.9	−0.22	−0.38	−0.06	0.0089
	Thailand	128	1.31	0.0 - 3.6	0.95	0.0 - 2.9	−0.36	−0.48	−0.23	<.0001

#### Asthma symptom score, days with awakening(s) during the night, inhalations of medications, asthma-control and asthma symptom-free days

Patients’ mean asthma symptom score was significantly reduced (P < 0.0001) while they were on treatment with budesonide/formoterol maintenance and reliever therapy. From run-in to treatment period, night- and day-time symptom score improved by 0.32 ± 0.54 (95% CI, 0.28 to 0.35) and 0.30 ± 0.52 (95% CI, 0.27 to 0.34), respectively. The percentage of days with awakening(s) due to asthma symptoms during the night was reduced by 11.09 ± 26.13% (95% CI, 9.34 to 12.85%; P < 0.0001), while the percentage of asthma-control and symptom-free days increased by 20.90 ± 34.40% (95% CI, 18.59 to 23.21%) and 23.89 ± 34.62% (95% CI, 21.56 to 26.21%), respectively (P < 0.0001).

During the run-in period, the number of inhalations (mean ± SD) of as-needed medications was 0.57 ± 0.88 inhalations during night-time and 0.65 ± 1.04 inhalations during day-time. The corresponding numbers of night-time and day-time inhalations during treatment period was 0.27 ±0.44 and 0.36 ± 0.56 inhalations, respectively. Along with that, the number of inhalations of as-needed reliever medications for night- and day-time was significantly reduced by 0.30 ± 0.82 (95% CI, 0.24 to 0.35) inhalations and 0.30 ± 0.97 (95% CI, 0.23 to 0.36) inhalations, respectively (P < 0.0001). Concurrently, the percentage of as-needed medication free days increased by 11.90 ± 44.65% (95% CI, 8.90 to 14.89%; P < 0.0001). During the period patients were on budesonide/formoterol maintenance and reliever therapy, only 1 patient (0.1%) had used SABA concomitantly for relief of symptoms.

#### Change in forced expiratory volume in one second (FEV_1_) from baseline level

**Figure 4:**
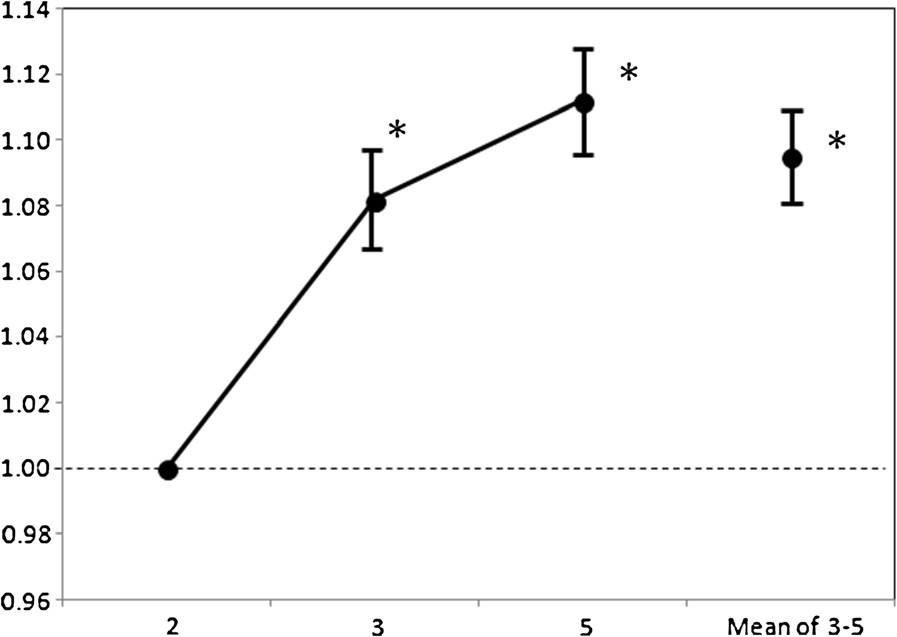
**G-mean FEV**_**1 **_**change from Visit 2 (baseline) and 95% CI.**

Patients’ lung function improved during treatment with budesonide/formoterol maintenance and reliever therapy. This was reflected by a significant increase in FEV_1_ G-mean value from baseline level as early as 4 weeks post-initiation of treatment (P < 0.0001), indicating an early onset in recovery of impaired lung function (Figure 4 and Table 5). At baseline, mean FEV_1_ (mean ± SD) was 1.96 ± 0.76 L and G-mean FEV_1_ (G-mean ± CV%) was 1.82 ± 40.78 L. During the 12 weeks of treatment, the overall increase in mean FEV_1_ from baseline was 0.17 ± 0.35 (95% CI, 0.15 - 0.20) L; the corresponding increase in G-mean FEV_1_ was 1.10 ± 19.33 (95% CI, 1.08 - 1.11) L (P < 0.0001).

**Table 5:** **Mean values of FEV**_**1 **_**at a regional level**

**Variable**	**Visit**	**Observed value**						**Change from visit 2 (baseline)**						**95% confidence interval**	
		**N**	**Mean**	**SD**	**Min**	**Median**	**Max**	**N**	**Mean**	**SD**	**Min**	**Median**	**Max**	**Lower**	**Upper**
FEV_1_ (L)	2	862	1.964	0.759	0.40	1.830	5.06	-	-	-	-	-	-	-	-
	3	836	2.110	0.779	0.55	1.985	4.91	836	0.152	0.374	−2.35	0.120	2.01	0.127	0.178
	5	805	2.160	0.767	0.52	2.060	5.15	805	0.199	0.393	−2.32	0.160	2.31	0.172	0.227
	Mean of 3 and 5	840	2.131	0.761	0.54	2.028	4.66	840	0.173	0.353	−2.34	0.130	2.03	0.149	0.197

### Assessment of impact on patients’ Quality of life

#### Change in AQLQ-S domains and overall scores from baseline (regional level)

During treatment with budesonide/formoterol maintenance and reliever therapy, patients’ overall AQLQ-S score improved by 0.70 ± 0.89 (95% CI, 0.64 to 0.76) from baseline level (P < 0.0001), demonstrating a significant overall improvement in patients’ quality of life across the region. Significant improvement was evident from as early as 4 weeks into the therapy, and the improvement continued throughout the treatment period (Table 6). Clinically important change (difference ≥ MID of 0.5) was evident from 8 weeks post-initiation of budesonide/formoterol maintenance and reliever therapy. While 52.9% of the patients experienced improvement in everyday functioning and well-being, only 5.1% of them experienced worsened conditions. Overall scores for symptoms, activity limitations, emotion function and response to environmental stimuli were likewise significantly increased during the treatment period (P < 0.0001), with significant improvement seen from as early as 4 weeks after initiation of treatment with budesonide/formoterol maintenance and reliever therapy.

**Table 6:** Mean AQLQ-S score at baseline and during treatment, at a regional level

**Variable**	**Visit**	**Observed value**						**Change from visit 2 (baseline)**						**95% confidence interval**		**Paired *t*-test**	**Wilcoxon signed rank test**
		**N**	**Mean**	**SD**	**Min**	**Median**	**Max**	**N**	**Mean**	**SD**	**Min**	**Median**	**Max**	**Lower**	**Upper**	**P-value**	**P-value**
Overall score	2	860	4.80	1.09	1.4	4.81	7.0	-	-	-	-	-	-	-	-	-	-
	3	838	5.31	1.00	1.8	5.41	7.0	837	0.51	0.89	−3.8	0.39	4.5	0.45	0.57	<.0001	<.0001
	4	811	5.54	0.99	1.7	5.71	7.0	810	0.74	0.95	−2.4	0.59	4.2	0.67	0.80	<.0001	<.0001
	5	804	5.67	0.98	1.9	5.84	7.0	803	0.87	1.04	−2.9	0.71	4.5	0.79	0.94	<.0001	<.0001
	Mean of 3-5	842	5.50	0.92	2.1	5.60	7.0	841	0.70	0.89	−2.2	0.55	4.3	0.64	0.76	<.0001	<.0001

#### Change in AQLQ-S domains and overall scores from baseline (country/area level)

Statistically significant improvement in overall AQLQ-S score was observed in all 5 participating countries/areas during treatment with budesonide/formoterol maintenance and reliever therapy (P < 0.0001 to P = 0.0002). Significant improvements from baseline levels were observed by 4 weeks of treatment (P < 0.0001 to P = 0.0252). The quantum of change however differed significantly between the populations studied in the various countries/areas (P < 0.0001); the change from baseline level was greatest in the study population in Indonesia, followed by that of those in India, China, Thailand and Taiwan. Clinically important change (difference ≥ MID of 0.5) in AQLQ-S score was observed only in Indonesia, Thailand, China and India. Significant improvements in individual domains (symptoms, activity limitations, emotional function and response to environmental stimuli) measured during treatment were also observed in all the study populations in the 5 countries/area.

### Safety

Amongst the 862 patients included in the safety analysis, 171 patients (19.8%) reported adverse events, and 12 patients (1.4%) reported serious adverse events. The most frequently reported adverse events were nasopharyngitis (3.4%), followed by upper respiratory tract infection (3.2%), and asthma exacerbation (1.4%). The majority (130 of 171) of the adverse events were of mild intensity. Thirteen patients (1.5%) had their study medication discontinued because of adverse events. Serious adverse events were uncommon, with only 15 events reported in 12 patients, 8 of which were considered severe in intensity, and one of them was life threatening. There were 3 cases of palpitations, the causality of which were judged by the investigator as possibly related to budesonide/formoterol maintenance and reliever therapy. Two of the serious adverse events, an asthma exacerbation and a myocardial infarction, resulted in death. Both deaths were however considered unrelated to budesonide/formoterol maintenance and reliever therapy.

## Discussion

This study documents the baseline and treatment outcome data collected during a 12-week budesonide/formoterol maintenance and reliever therapy in 862 Asian patients with partially controlled or uncontrolled asthma, treated in a real-life clinical practice setting. During treatment with budesonide ⁄ formoterol maintenance and reliever therapy, administered one inhalation, twice daily for maintenance therapy, plus as-needed for reliever medication, patients whose asthma conditions were not adequately controlled while on their previous medications had their asthma conditions significantly improved.

At baseline, the overall ACQ-5 of patients assessed in the region was 1.62 ± 1.00, indicating generally poorly controlled asthma in the study population. Following the 12-week budesonide/formoterol maintenance and reliever therapy, the overall ACQ-5 score improved significantly to 0.92 ± 0.85 (P < 0.0001). Significant relief of asthma symptoms was observed as early as 4 weeks into budesonide⁄formoterol maintenance and reliever therapy, while clinically important improvement was observed by 8 weeks of treatment, indicating early improvement in asthma control after initiation of the therapy. The improvement in asthma control was sustained throughout the 12-week treatment period.

Along with improvements in asthma control, as-needed use of reliever medication was significantly reduced; patients experienced more reliever medication-free days during budesonide/formoterol maintenance and reliever therapy than during the run-in period (P < 0.0001). Patients’ diary records showed asthma symptoms and night awakenings due to the symptoms were both significantly reduced during the treatment period (P < 0.0001). The percentage of both the asthma control days and symptom-free days were significantly increased during budesonide/formoterol maintenance and reliever therapy (P < 0.0001). In addition, FEV_1_ levels measured before and after budesonide/formoterol maintenance and reliever therapy indicated a significant improvement in lung function from the baseline level during the therapy (P < 0.0001). As with the patient-reported outcomes described above, improvement in lung function, as measured by FEV_1_, were apparent from as early as 4 weeks after initiation of budesonide/formoterol maintenance and reliever therapy and was sustained throughout the 12-week treatment period.

Together with better asthma control, patients’ quality of life, as assessed by the AQLQ-S overall and domain scores, improved significantly during budesonide/formoterol maintenance and reliever therapy. At the regional level, the overall changes in AQLQ-S scores from baseline to treatment period were both statistically significant and clinically important, with about half of the patients treated showing improvements in asthma control, as well as in their everyday functioning and well-being.

At the country/area level, although the improvements in asthma control and quality of life, as indicated by the change in ACQ-5 and AQLQ-S scores from baseline to treatment period, were statistically significant in all 5 participating countries/areas, clinically important change in the ACQ-5 score was observed in China, India and Indonesia, but not in Thailand and Taiwan. Similarly, clinically important change in AQLQ-S score was evident in Indonesia, Thailand, China and India, but not in Taiwan. Although, by 4 weeks of budesonide/formoterol maintenance and reliever therapy, the changes in both ACQ-5 and AQLQ-S scores already reached statistical significance in all countries, clinically important difference in ACQ-5 score from baseline was only achieved in China and Indonesia, while that of AQLQ-S score was only achieved in China, India and Indonesia.

The reason behind the significant inter-country/area difference in the treatment outcomes and the time lag in achieving clinically important improvements in certain countries is not clear. Although the wide difference in sample size between the countries might have skewed the statistics, the impact of sample size is probably minimal, judging from the fact that Indonesia, which has the smallest sample size, had results that showed a most pronounced improvement in asthma control in its study population. It is possible that inter-country difference in clinical management of asthma, which might also result in differences in patients’ baseline variables, might have affected the quantum of change observable post-treatment. Moreover, outcome data could, at least in part, be affected by the variation in disease epidemiology in the different countries/areas, and the dissimilarity in the way symptoms are perceived across geographical and cultural barriers. In a real-life study setting such as this one, few, if any, potential confounders could be controlled.

While the design of the study does not allow for any conclusions regarding the effect of budesonide/formoterol maintenance and reliever therapy, it being a within-group comparison, the results are in line with what has been previously seen [23-26]. Owing to the study design, the beneficial effect may be considered as a result of simply step-up therapy. However, patients in majority (60.9%) were already on ICS plus LABA before entry. Further improvement of symptoms and lung function may be attributed to the SMART regimen. In addition, the improvements in symptom control, lung function, and reduction in as-needed reliever medications were comparable to those previously reported in earlier studies [24,27].

In terms of safety, budesonide/formoterol administered one inhalation, twice daily as maintenance therapy, plus as-needed as symptom reliever, was well tolerated by patients during the 12-week treatment period. Only 19.8% of the treated patients reported adverse events, and 1.4% of patients reported serious adverse events during the study. The majority of the reported adverse events were of mild intensity. Only 1.5% of the patients presenting with adverse events had their study medication discontinued because of the adverse events. There were 3 cases of palpitations possibly related to budesonide/formoterol maintenance and reliever therapy. Two adverse events (0.2%) resulted in deaths, but both were considered unrelated to budesonide/formoterol maintenance and reliever therapy. There was no evidence of overuse of budesonide/formoterol as a reliever medication. Observations concerning safety of the treatment was in line with the results of other studies on budesonide/formoterol maintenance and reliever therapy [28,29], and we did not identify any new safety-related issue with the budesonide/formoterol maintenance and reliever therapy during the 12-week treatment period.

In summary, the results are in line with previous findings that a single inhaler therapy for asthma with combined budesonide and formoterol as both maintenance therapy and reliever provided better control of asthma than conventional combination inhalers. Budesonide/formoterol, administered one inhalation twice a day as maintenance therapy and as-needed, was well tolerated.

## Conclusions

During treatment of inadequately controlled asthmatic patients with budesonide/formoterol maintenance and reliever therapy, significant improvement in patients’ asthma control and reductions in asthma symptoms and as-needed medication use was observed. Patients’ quality of life was improved and the treatment was well tolerated.

### Appendix I

List of Independent Ethics Committees/Institutional Review Boards

Ethics Committee of 1st Affiliated Hospital of Guangzhou Medical College

No. 151, West Yanjiang Road, Guangzhou

Guangdong Province, 510120, China

Ethics Committee of China-Japan Friendship Hospital

East Street of Ying Hua Yuan, Chaoyang District

Beijing, 100029, China

Peking University Third Hospital State Drug Clinical Study Ethics Committee

49 North Garden Rd., Haidian District

Beijing 100191, China

Peking Union Medical College Hospital Drug Clinical Trial Ethics Committee

No. 1 Shuaifuyuan Wangfujing, Dongcheng district

Beijing 100730, China

Ethics Committee of Beijing Chaoyang Hospital, Capital Medical University

Gongtinan Road, Chaoyang District

Beijing, 100020, China

The First Hospital of China Medical University Medical Ethics Committee

No. 155, North Street of Nanjing, Heping District

Shenyang 110001, China

Ethic Committee of Qingdao Medical College Affiliated Hospital

No. 16 Jiangsu Road

Qingdao, 266003, China

Ethics Committee of Second Affiliated Hospital

School of Medicine, Zhejiang University

88 Jiefang Road

Hangzhou 310009, China

Ethics Committee of Affiliated Shao Yifu Hospital

School of Medicine, Zhejiang University

3 East Qingchun Road

Hangzhou 310016, China

Shanghai Changzheng Hospital

No. 415, Fengyang Road

Shanghai 200003, China

Ethics Committee of the Affiliated Nanfang Hospital, Southern Medical University

No. 1838, North Guangzhou Road, Baiyun Dinstrict

Guangzhou, Guangdong Province, China

Ethics Committee of Guangdong General Hospital

No. 106, Zhongshan 2nd Road, Guangzhou

Guangdong Province, China 510080

Ethics Committee of Tongji Medical School of Huazhong University of Science and Technology

No. 13, Hangkong Road, Wuhan

Hubei Province, China

Ethics Committee of Tongji Medical School of Huazhong University of Science and Technology.

No. 13, Hangkong Road, Wuhan

Hubei Province, China

Ethics Committee of Fuzhou General Hospital of Nanjing Command

No. 156, Xi Er Huan North Road, Gulou District, Fuzhou

Fujiang Province, 350025, China

Ethics Committee of The Second Xiangya Hospital Of Central-South University

No. 139 Middle Renmin Road, Changsha

Hunan, China 410011

Ethics Committee of Huaxi Hospital

No. 37 Wainanguoxue Road

Chengdu, 610041, China

Henan Provincial Peoples Hospital Drug Clinical Trial Ethics Committee

No. 1, The fifth Road of Wei

Zhengzhou 450003,

Henan Province, China

NanjingDrum Tower Hospital

No. 321, Zhongshan Road

Nanjing, 210008, China

Ethics Committee of Shanghai Jiaotong University School of Medicine

Affiliated XinHua Hospital

1665, Kongjiang Road, Yangpu District

Shanghai, 200092, China

Ethics Committee of The First Affiliated Hospital of Henan Traditional Medicine College

No. 19, Renmin Road, Zhengzhou 450000

Henan Province, China

The Fortis Hospital Institutional Review Board (FHIRB)

B-22, Sector-62, Noida 201 301

India

Independent Ethics Committee

B-1/170, Sector-G, Aliganj

Lucknow, India

Safe Search Independent Ethics Committee

Sidhhachal Complex, Nr. Doordarshan Kendra

Thaltej, Ahmedabad 380054

Gujarat, India

Sterling Hospital Ethics Committee, Sterling Hospital Road

Memnager, Ahmedabad 380052

Gujarat, India

Institutional Ethics Committee – Global Hospitals & Health City

439, Cheran Nagar, Perumbakkam

Chennai 600 100, India

Institutional Ethics Committee

SRI Ramachandra University

India

Ethics Committee, Bengaluru Allergy Immunology Research Foundation India

No. ½, 1st Cross, CSI Compound, Mission Road

Bangalore 560027, India

SAMEEKSHA Independent Ethics Committee

H. No. 6-3-609/15, Anand Nagar Colony, Khairatabad

Hyderabad500 004, Andhrapradesh, India

CEREBRAL Independent Review Board

301/A. Lenaine Estate, Abids

Hyderabad 500 001, India

Institutional Ethics Committee, Coimbatore Chest Clinic

M.S.S. Memorial Building, OPP. Savitha Hall, 8, D.B. Road

R.S. Puram, Coimbatore 641 002, India

Bangalore Central Ethics Committee

No. 1423, Kullappa Layout, Kullappa Circle

St Thomas Town P.O., Bangalore 560084

Karnataka, India

Apex Independent Ethics Committee

3, Old Agrawal Nagar, Indore 452 001

Madhya Pradesh, India

Universitas Indonesia Fakultas Kedokteran

Jalan Salemba Raya No. 6 Jakarta Pusat

Pos Box 1358, Jakarta 10430

Indonesia

Institutional Review Board, Chung Shan Medical University Hospital

No. 110, Sec. 1, Chien-Kuo N. Road,\

Taichung, Taiwan 40201

Institutional Review Board of Tri-Service General Hospital

National Defense Medical Center

No. 325, Sec.2, Cheng-Kung Rd

Neihu 11490 Taipei, Taiwan

Mackay Memorial Hospital, Institutional Review Board Approval of Clinical Trial

92, Sec. 2, Chungshan N. Rd

Taipei 10449, Taiwan

Institutional Review Board, Chiayi Christian Hospital

539 Jhongsiao Rd

Chia-Yi City, Taiwan 60002

Institutional Review Board, Yuan’s General Hospital

No.162 Cheng Kung 1st Road

Kaohsiung 80249, Taiwan

Institutional Review Board, Chung Shan Medical University Hospital

No.110, Sec. 1, Chien-Kuo N. Road

Taichung, Taiwan 40201

Institutional Review Board, Taoyuan General Hospital

Department of Health, Executive Yuan

No.1492, Zhongshan Rd

Taoyuan City 330, Taiwan

Mackay Memorial Hospital, Institutional Review Board Approval of Clinical Trial

92, Sec. 2, Chungshan N. Rd

Taipei 10449, Taiwan

Institutional Review Board, National Taiwan University Hospital

No.7 Chung San South Road

Taipei City, Taiwan

Institutional Review Board, Cheng Ching General Hospital

No. 118, Sec. 3 Jung Gang Rd

Taichung 407, Taiwan

Institutional Review Board, E-DA Hospital

No 1, E-Da Road, Jiau-Shu Tsuen, Yan Chau Shiang

Kaohsiung, County, Taiwan

Institutional Review Board, Faculty of Medicine, Chulalongkorn University

1873 Rama 4 Road, Patumwan

Bangkok 10330, Thailand

Research Ethics Committee, Police General Hospital

492/1, Rama 1 Rd, Wangmai, Patumwan

Bangkok, 10330, Thailand

Documentary Proof of Ethical Clearance Committee on Human Rights Related to Researches Involving Human Subjects

Faculty of Medicine, Ramathibodi Hospital, Mahidol University

Rama VI Road

Bangkok 10400, Thailand

Documentary Proof of Institutional Review Board, Royal Thai Army, Medical Department

317 Rajavithi Road, Rajathevee

Bangkok 10400, Thailand

Research Ethics Committee, Nopparat Rajathanee Hospital

Clinical Research Center, 679 Nopparat-Rajathanee Hospital

Ramintra Street, Kanayaw

Bangkok 10230, Thailand

Institutional Review Board, Maharat Nakhon Ratchasima Hospital

49, Chang Phueang District

Nakhon Ratchasima Province, 30000, Thailand

## Competing interests

Nanshan Zhong: involved in COPD research sponsored by Boehringer Ingelheim and Asthma research from Novartis. Tzu-Chin Wu: involved in COPD research sponsored by Boehringer Ingelheim and a lung cancer study. JiangTao Lin, Parthiv Mehta, Pintip Ngamjanyaporn, Faisal Yunus: declare that they have no competing interest.

## Authors’ contributions

NZ was the international principal investigator of the study. He contributed to the design of the study, recruitment of patients, interpretation of the study results, development and approval of the final content of the manuscript, and made decision to submit the manuscript for publication. JL, PM, PN, TW and FY are national principal investigators for China, India, Thailand, Taiwan and Indonesia, respectively. They all contributed to the recruitment of patients, interpretation of the study results, development and approval of the final content of the manuscript. All authors made decision to submit the manuscript for publication. All authors read and approved the final manuscript.

## Pre-publication history

The pre-publication history for this paper can be accessed here:

http://www.biomedcentral.com/1471-2466/13/22/prepub
